# Copper‐Driven Epithelial Barrier Disruption: A Novel Mechanism of COPD Acute Exacerbations Mediated by the TNF‐α/ATP7A Axis

**DOI:** 10.1002/advs.202519533

**Published:** 2026-09-27

**Authors:** Xinru Xiao, Yongzhe Hao, Ziqi Ding, Zhipeng Wang, Yujia Shi, Yanhua Huang, Chunjian Qi, Qian Zhang

**Affiliations:** ^1^ Department of Respiratory and Critical Care Medicine The Second People's Hospital of Changzhou The Third Affiliated Hospital of Nanjing Medical University Changzhou China; ^2^ Department of Central Laboratory The Second People's Hospital of Changzhou The Third Affiliated Hospital of Nanjing Medical University Changzhou China

**Keywords:** acute exacerbations of chronic obstructive pulmonary disease, ATPase copper transporting alpha, copper homeostasis, epithelial barrier disruption, Tumor necrosis factor‐α

## Abstract

Elevated copper levels are associated with chronic obstructive pulmonary disease (COPD) and susceptibility to acute exacerbations of COPD (AECOPD), but the underlying mechanisms remain unclear. This study aimed to investigate the association between copper levels and AECOPD and elucidate the underlying mechanisms. A cross‐sectional analysis of NHANES 2011–2012, a hospital‐based case‐control study enrolling 30 AECOPD patients and 30 healthy controls, and a one‐year prospective follow‐up of COPD patients demonstrated that higher serum copper levels were associated with more frequent exacerbations and earlier recurrence. Integrated studies using copper‐manipulated COPD mice, zebrafish, lung organoids, and BEAS‐2B cells, together with bulk and single‐cell RNA sequencing, colorimetric copper quantification, ELISA‐based tumor necrosis factor‐α (TNF‐α) measurement, oxidative stress and mitochondrial functional analyses, and FITC‐dextran permeability assays, showed that excess copper induced mitochondrial oxidative stress, tight‐junction loss, and epithelial barrier dysfunction. Mechanistic analyses revealed that TNF‐α suppressed ATPase copper‐transporting alpha (ATP7A) transcription through competition between NF‐κB and CREB1 for the shared coactivator CBP, thereby promoting intracellular copper accumulation. ATP7A restoration alleviated epithelial injury, whereas ATP7A silencing abolished the improvement conferred by anti‐TNF‐α therapy in COPD mice. These findings identify the copper–TNF‐α/ATP7A axis as a mechanism underlying AECOPD and a potential therapeutic target.

## Graphical abstract

This study reveals a copper‐driven mechanism of COPD acute exacerbations mediated by the TNF‐α/ATP7A axis. TNF‐α suppresses the copper transporter ATP7A via competitive NF‐κB/CREB1/CBP signaling, resulting in intracellular copper accumulation, mitochondrial oxidative stress, and epithelial barrier disruption.

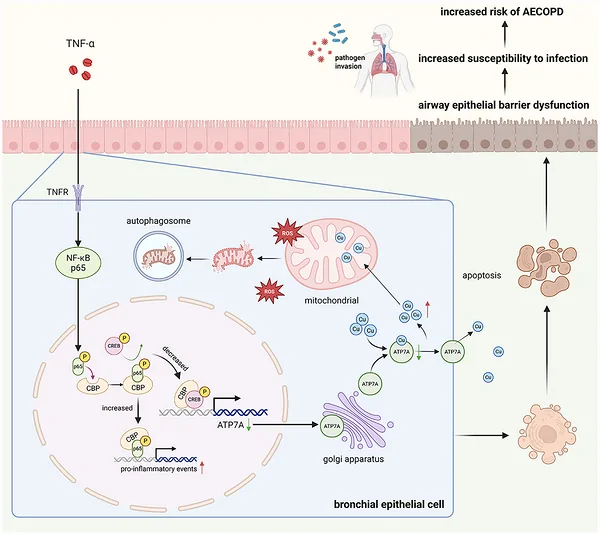

## 1 Introduction

Chronic obstructive pulmonary disease (COPD) remains a significant global health burden, with projections indicating that it will become the third leading cause of mortality worldwide by 2030. Acute exacerbations of COPD (AECOPD) play a critical role in disease progression and are closely associated with adverse clinical outcomes. Environmental factors, particularly tobacco smoke and air pollution, are well‐documented contributors to the pathogenesis of COPD [1]. Inhalation of tobacco smoke and air pollutants is widely recognized as a major trigger of airway epithelial barrier dysfunction [2]. The airway epithelium functions not only as a physical barrier but also as a key immune coordinator [3]. When damaged by smoke or pollutants, epithelial cells release alarmins, which drive the neutrophilic and eosinophilic inflammation characteristic of COPD. These alarmins further disrupt epithelial tight and adherens junctions and promote a pro‐inflammatory cascade, thereby exacerbating disease progression [4].

Tobacco contains various toxic metals that are released into smoke during combustion and subsequently accumulate in the lung tissues of smokers [5, 6]. Moreover, exposure to air pollutants—particularly fine particulate matter (PM_2.5_)—has been consistently associated with an increased risk of developing COPD [7, 8, 9]. Metals constitute a key component of the harmful substances present in PM_2.5_ [10, 11], primarily originating from anthropogenic sources such as fuel combustion, brake and rail wear, and engine abrasion [12]. Importantly, these ambient metals are increasingly recognized for their potential to disrupt the homeostasis of essential trace elements in the body, thereby exacerbating COPD pathogenesis and contributing to more severe health consequences.

Copper (Cu) ranks among the top three metals present in cigarette tobacco and smoke [5, 13] and is prevalent in air and water pollution [14]. Pulmonary copper homeostasis is maintained by a multi‐layered regulatory system. High‐affinity copper uptake is mediated by copper transporter 1 (CTR1), while intracellular delivery to specific enzymes is facilitated by chaperones including antioxidant 1 copper chaperone (ATOX1), copper chaperone for superoxide dismutase (CCS), and cytochrome c oxidase copper chaperone 17 (COX17). These chaperones ensure metallation of key cuproenzymes, such as mitochondrial cytochrome c oxidase and Cu/Zn‐superoxide dismutase, maintaining efficient mitochondrial respiration, ATP generation, and antioxidant defense. Copper export and detoxification are then controlled by ATPase copper transporting alpha/beta (ATP7A/B), which traffic between the trans‐Golgi network and vesicles to supply cuproenzymes or remove excess copper [15, 16, 17]. Dysregulation of these transporters and chaperones, as observed in fibrotic diseases, disrupts copper balance, impairs enzyme activity and mitochondrial ATP generation, increases oxidative stress, and contributes to pathological progression [18]. These processes also play a critical role in the pathogenesis of COPD. Although elevated copper levels have been consistently reported in patients with COPD [7, 19, 20], emerging evidence suggests that copper dysregulation may also be associated with acute exacerbations. Serum copper levels have been reported to be higher in patients with AECOPD than in those with stable COPD or healthy controls [21]. However, the precise molecular mechanisms underlying copper‐mediated lung injury and increased susceptibility to AECOPD remain poorly defined. The dual nature of copper further complicates mechanistic understanding, as both deficiency and excess can promote disease through distinct pathways [22], thereby hindering the development of targeted therapeutic strategies aimed at restoring copper homeostasis. Although nutritional recommendations and serum reference intervals for copper are available, there is currently no universally accepted single copper threshold that defines optimal biological function in the airway epithelium. In chronic inflammatory lung diseases such as COPD, the key pathogenic issue may be disruption of copper homeostasis rather than absolute exposure alone. Therefore, identification of key regulatory nodes—including copper transporters, inflammatory mediators, and cuproptosis‐related factors—may enable more precise interventions to reconcile copper's essential and pathological roles, address the complexity of COPD, and ultimately improve clinical outcomes.

In this study, we integrated epidemiological analyses of the National Health and Nutrition Examination Survey (NHANES) cohort and clinical follow‐up data with preclinical models, including copper‐manipulated COPD mouse models, zebrafish, lung organoids, and human bronchial epithelial cells. Transcriptomic and single‐cell analyses were employed to identify regulatory pathways, followed by functional validation using gene knockdown and overexpression approaches. Collectively, these strategies provide a comprehensive assessment of copper's role in AECOPD, linking clinical observations with underlying molecular mechanisms.

## 2 Methods

### 2.1 Human Cohort

We used data from the 2011–2012 NHANES. Participants were excluded if they had a post‐bronchodilator forced expiratory volume in 1 s (FEV_1_)/forced vital capacity (FVC) > 0.7, missing serum copper data, or were younger than 18 years of age. Acute exacerbation was defined as wheezing or hospitalization within the past 12 months.

Sixty participants (30 healthy controls and 30 patients with AECOPD) were enrolled at Changzhou No.2 People's Hospital between August 2023 and August 2024. AECOPD was diagnosed in accordance with the 2024 Global Initiative for Chronic Obstructive Lung Disease (GOLD) criteria. Exclusion criteria included pregnancy, concurrent lung diseases (e.g., bronchiectasis, asthma, or malignancy), and systemic inflammatory conditions. Serum copper levels were measured using a copper detection kit (Mlbio, Shanghai, China) according to the manufacturer's instructions. Pulmonary function was assessed using a spirometer (Jaeger, Bavaria, Germany). Flow–volume curves were continuously monitored, and FVC, FEV_1_, FEV_1_/FVC, and their predicted values were recorded. For patients with AECOPD, pulmonary function data were obtained from the most recent post‐bronchodilator spirometry performed during a clinically stable period within 6 months before enrollment.

### 2.2 Mouse COPD Model

Male C57BL/6J mice (6‐8 weeks old; Cavens) were housed under controlled conditions (22°C ± 0.5°C, 50% ± 5% humidity) with a 12‐h light/dark cycle. COPD was induced by passive cigarette smoke (CS) exposure (60 min, twice daily, 5 days per week) for 12 weeks in all experimental groups. For copper‐related interventions, mice received either copper sulfate (CuSO_4_, Sigma–Aldrich, Darmstadt, Germany, 30 mg/kg/day, ≥ 99.99% trace metal basis, total trace metal impurities ≤ 100 ppm) [23] as a copper challenge by gavage prior to CS exposure, tetrathiomolybdate (TTM, Sigma–Aldrich, Darmstadt, Germany, 0.7 mg/day) [24] by gavage for copper chelation (n = 5 per group), or CuSO_4_ (50 mg/L) administered in drinking water [25]. Endotoxin testing of the CuSO_4_ solutions confirmed concentrations below the detection limit (< 0.125 EU/mL). All treatments were initiated at week 9. In a separate experiment, another group of mice subjected to the same 12‐week CS‐induced COPD model was intranasally inoculated with 50 µL of H1N1 virus suspension (titer: 5.6 × 10^6^ TCID_50_/mL) after completion of CS exposure at week 12 (n = 5 per group). Pulmonary function was assessed seven days after viral inoculation. To evaluate the role of ATP7A in anti‐tumor necrosis factor‐α (TNF‐α) therapy, an additional group of mice received intratracheal administration of AAV6‐ATP7A‐shRNA or control virus at week 9 (GenePharma, Suzhou, China, 50 µL, 1 × 10^13^ vp/mL), followed by biweekly intraperitoneal injections of infliximab (5 mg/kg) for 4 weeks during the CS exposure period (n = 5 per group). Mice subsequently underwent micro‐CT scanning under isoflurane anesthesia, and images were analyzed using RenderQuick software. Pulmonary function was then assessed using an EMMS Pulmonary Function Test (FM, UK), including measurements of FEV_25_, FVC, PEF, and FEF_10_. Bronchoalveolar lavage fluid (BALF), serum, and lung tissues were collected from each group for subsequent analyses.

For pathological staining, left lung lobes were fixed in 4% paraformaldehyde and subsequently embedded in paraffin. Paraffin sections were deparaffinized and subjected to hematoxylin and eosin (H&E), periodic acid‐Schiff (PAS), and Masson's trichrome (MASSON) staining. Immunohistochemistry (IHC) for ATP7A (1:100, Affinity, Jiangsu, China) and terminal deoxynucleotidyl transferase dUTP nick‐end labeling (TUNEL) assays (Beyotime, Shanghai, China) were performed according to standard protocols. For transmission electron microscopy (TEM), specimens were pre‐fixed with 2.5% glutaraldehyde, rinsed with PBS, post‐fixed in 1% osmium tetroxide, dehydrated through graded ethanol, infiltrated with embedding medium/acetone, embedded, sectioned, and double‐stained with lead citrate and 50% uranyl acetate. Tissue oxidizing activity was assessed by dihydroethidium (DHE) staining. Fresh tissue samples were embedded in OCT compound and snap‐frozen to prepare cryosections, which were then incubated with 10 µM DHE (MCE, NJ, USA). Fluorescence intensities of ATP7A, occludin, TUNEL, and DHE were quantified using ImageJ and expressed as the ratio of positive staining area to total tissue area. TNF‐α levels in mouse BALF were measured by ELISA (Elabscience, Wuhan, China). Malondialdehyde (MDA) levels were determined using an MDA detection kit (Beyotime, Shanghai, China). Copper levels in mouse serum, BALF, and lung tissue were quantified using a copper colorimetric assay kit (E‐BC‐K300‐M, Elabscience, Wuhan, China), with lung tissue copper concentrations normalized to total protein content.

### 2.3 Organoid Culture

Mouse primary cells were isolated, resuspended in organoid medium (AimingMed, Hangzhou, China), mixed with Matrigel (Corning, NY, USA) at a 1:2 ratio (50 µL per well), and allowed to solidify in 24‐well plates. Cultures were maintained in MasterAim medium at 37°C with 5% CO_2_. H&E staining was performed to evaluate the structure and morphology of the organoids. To characterize the major cellular composition of lung organoids, flow cytometry analysis was performed using fluorescence‐conjugated antibodies against CD45, CD326, CD140a, and CD31 (all from Biolegend, CA, USA) following standard protocols. Cell‐type identification was defined as follows: CD45^+^ for immune cells, CD45^−^CD326^+^ for airway epithelial cells, CD45^−^CD140a^+^ for lung fibroblasts/mesenchymal cells, CD45^−^CD31^+^ for pulmonary vascular endothelial cells, and CD45^−^CD326^−^CD140a^−^CD31^−^ for other unclassified cell types. Organoids with diameters greater than 100 µm were treated with CuCl_2_ (Sigma–Aldrich, Darmstadt, Germany, 1.57–50 µm) for 72 h, followed by measurements of ATP (Beyotime, Shanghai, China) and reactive oxygen species (ROS, Beyotime, Shanghai, China) levels. Organoids exposed to 50 µm CuCl_2_ were further subjected to immunofluorescence (IF) staining for occludin, a key tight junction marker, and quantitative real‐time polymerase chain reaction (RT‐qPCR) to assess occludin mRNA expression, thereby comprehensively evaluating tight junction integrity.

### 2.4 Cell Assays

BEAS‐2B cells were cultured in DMEM supplemented with 10% fetal bovine serum (FBS, Gibico, NY, USA) and 1% penicillin/streptomycin at 37°C with 5% CO_2_. THP‐1 cells were cultured in RPMI‐1640 medium supplemented with 10% FBS, 0.05 mM β‐mercaptoethanol, and 1% penicillin/streptomycin. THP‐1 cells were stimulated with 100 nm phorbol 12‐myristate 13‐acetate (PMA, MCE, NJ, USA) for 24 h prior to subsequent treatment. Cells were treated with CuCl_2_ (10–100 µm) for 24 h, or with 50 ng/mL of various cytokines, including TNF‐α, interleukin‐1β (IL‐1β), IL‐6, IL‐8, IL‐10, and IL‐17A (all from PeproTech, NJ, USA), either alone or in combination with 10 µm CuCl_2_ for 24 h. For cycloheximide (CHX) assays, following stimulation with 50 µM CuCl_2_, the culture medium was replaced with complete medium containing 100 µg/mL CHX (MCE, NJ, USA). ATP7A subcellular localization was examined, or protein samples were harvested at the indicated time points. ATP7A expression was silenced using siRNA or overexpressed via pcDNA3.1‐ATP7A plasmid transfection (All from GenePharma, Suzhou, China). Rescue experiments were performed by pretreating cells with 20 µm TTM for 2 h prior to exposure to CuCl_2_ combined with ATP7A‐siRNA.

Cell viability was assessed using the Cell Counting Kit‐8 (CCK‐8) assay (Dojindo, Tokyo, Japan). Apoptosis was analyzed using the Annexin V‐FITC/PI apoptosis detection kit (Vazyme, Nanjing, China) and flow cytometry. ROS production was measured using DCFH‐DA or MitoSOX Red (Beyotime, Shanghai, China) by flow cytometry. Mitochondrial membrane potential (Δψm) was evaluated using the JC‐1 assay kit (Beyotime, China). Oxygen consumption rate (OCR) was measured in real time using a Seahorse XF Analyzer (CA, USA) with sequential injections of oligomycin (1 µm), FCCP (1 µM), and rotenone (1 µm). TNF‐α levels were quantified using ELISA kits (Elabscience, Wuhan, China).

IF assays were performed on cells seeded in 35 mm glass‐bottom dishes. Primary antibodies against ATP7A (1:200), LC3 (1:500, Proteintech, Wuhan, China), CBP (1:200, CST, MA, USA), p65 (1:500, Proteintech, Wuhan, China), and occludin (1:500, Proteintech, Wuhan, China) were applied, followed by fluorescent secondary antibodies and DAPI nuclear staining. Mito‐Tracker Green (Beyotime, Shanghai, China) and DiO (Beyotime, Shanghai, China) were used to label mitochondria and cell membranes, respectively. For TEM, cells were fixed, dehydrated, embedded, sectioned, and stained to examine ultrastructural features.

Western blotting was performed using cell lysates, with primary antibodies against ATP7A (1:1000), c‐myc (1:1000, Proteintech, Wuhan, China), p65 (1:1000), p‐p65 (1:1000, Proteintech, Wuhan, China), and GAPDH (1:1000, Proteintech, Wuhan, China). Co‐immunoprecipitation (Co‐IP) was conducted using IP lysis buffer, with immune complexes captured by Protein A magnetic beads and analyzed by western blotting using antibodies against p‐p65 or p‐CREB (1:1000, CST, MA, USA). Intracellular copper levels were quantified using a copper assay kit (E‐BC‐K775‐M, Elabscience, Wuhan, China). Epithelial permeability to macromolecules was assessed using the 40 kDa FITC‐dextran trans‐monolayer flux assay [26, 27, 28].

Dual‐luciferase reporter assays were performed to validate CREB1 binding to the ATP7A promoter: The wild‐type ATP7A promoter, as well as ATP7A promoter constructs with deletion of CREB1‐binding sites 4 and 6, and CREB1 cDNA were cloned into the pGL4 and pCDNA3.1 vectors (all from GenePharma, Suzhou, China), respectively. These constructs were transfected into 293T cells, and luciferase activity was measured 48 h post‐transfection. Chromatin immunoprecipitation followed by quantitative PCR (ChIP‐qPCR) was performed using a commercial kit (BaiWei, Guangzhou, China) with an anti‐CREB antibody or isotype IgG as a control, followed by qPCR analysis to quantify enrichment of the ATP7A promoter.

### 2.5 RNA Sequencing and Data Analyses

#### 2.5.1 Single‐Cell RNA Sequencing and Data Analyses

Single‐cell RNA‐seq analysis was performed by NovelBio using the NovelBrain Cloud platform. Raw reads were trimmed and quality‐filtered using fastp, and cell barcodes and unique molecular identifiers (UMIs) were processed using UMI‐tools. Clean reads were aligned to the mouse genome (mm10, Ensembl version 100) using STAR, and UMI count matrices were generated. Cells expressing fewer than 200 genes or with mitochondrial UMIs exceeding 20% were excluded from downstream analysis. Subsequent analyses were conducted using the Seurat (v4.1.1) pipeline, including log‐normalization, regression of confounding variables, principal component analysis based on 2000 highly variable genes, and dimensionality reduction using t‐SNE or UMAP based on the first 10 principal components. Graph‐based clustering was performed, and marker genes for each cluster were identified using the FindAllMarkers function (Wilcoxon rank‐sum test; thresholds: log2 fold change > 0.25, *p* < 0.05, and min.pct > 0.1). Clusters corresponding to the same cell type were merged and re‐clustered. Gene Ontology (GO) and Kyoto Encyclopedia of Genes and Genomes (KEGG) enrichment analyses were performed using clusterProfiler, and the significance of cell–cell communication was calculated based on intercellular interaction pairs.

#### 2.5.2 Bulk RNA Sequencing and Data Analyses

Total RNA was isolated using TRIzol, and 1 µg of RNA was used for library construction: including mRNA enrichment, fragmentation at 94°C, double‐stranded cDNA synthesis, end repair, A‐tailing, adapter ligation, size selection (300–500 bp), and 12 cycles of PCR amplification. Libraries were sequenced after library quality control assessment. Differentially expressed genes (DEGs) were identified using the Limma package in R, with a corrected *p* value < 0.05 and |log2 fold change| ≥ 1 as significance thresholds. GO and KEGG enrichment analyses, as well as gene set enrichment analysis (GSEA), were conducted using clusterProfiler.

### 2.6 Bioinformatics

Bulk RNA sequencing dataset GSE106986 (5 healthy and 14 COPD human lung tissue samples) and Single‐cell RNA sequencing dataset GSE173896 (7 healthy and 9 COPD human lung tissues samples) were retrieved from the Gene Expression Omnibus database for subsequent analyses. Copper‐related targets and COPD therapeutic targets were identified using the Comparative Toxicogenomics Database (CTD), Online Mendelian Inheritance in Man, DisGeNET, and GeneCards databases. Potential transcription factors regulating ATP7A were predicted using ALGGEN PROMO, Cistrome DB, hTFtarget, and UCSC JASPAR, and overlapping candidates were identified using Venn diagram analysis. Molecular docking of CREB1 with the ATP7A promoter was performed using AlphaFold3 in combination with Schrödinger's Protein Preparation Wizard. Classical molecular dynamics simulations were subsequently conducted using Desmond for 100 ns. Dynamic parameters, including root mean square deviation, root mean square fluctuation, radius of gyration, and solvent‐accessible surface area, as well as binding free energy calculated by the MM‐GBSA method, were analyzed. In silico ATP7A overexpression was performed in epithelial cells from the COPD group of GSE173896 using scTenifoldNet. The raw UMI count matrix was filtered to retain genes expressed in ≥ 5% of cells. ATP7A overexpression was simulated by adding 1 transcript count to ATP7A in all COPD epithelial cells, while keeping all other genes unchanged. Gene regulatory networks were constructed for the original and ATP7A‐perturbed matrices and denoised by tensor decomposition using nNet = 10, nCells = 200, nComp = 3, and K = 3. The two networks were compared by manifold alignment, and differential regulation scores were calculated based on embedding distances. Significant regulatory changes were defined as FDR < 0.05 and used for downstream pathway enrichment analysis.

### 2.7 Zebrafish Experiments

Zebrafish were included as an ideal model organism for respiratory epithelial injury research to rapidly validate dose–response relationships, verify the direct effect of copper as a single factor in vivo, and compensate for the limitations of the mouse complex COPD model and in vitro experiments. Based on this design, the following experimental procedures were performed. Three‐month‐old wild‐type AB strain zebrafish were purchased from Nanjing YSY Biotech and acclimated for two weeks prior to experimentation. Zebrafish were exposed to CuSO_4_ at concentrations of 0, 0.1, 0.2, 0.4, 0.8, and 1.6 µg/L for 3 days, with eight fish per group. Following exposure, zebrafish were anesthetized and stained with a ROS fluorescent probe. Subsequently, gill tissues were dissected and subjected to H&E staining to evaluate structural integrity.

### 2.8 Statistics

All statistical analyses were performed using GraphPad Prism 9. Data are presented as mean ± standard error of the mean (SEM). Statistical comparisons between two groups were performed using independent sample t‐tests, while comparisons among multiple groups were conducted using ANOVA followed by Tukey's HSD test. Univariate and multivariate Cox proportional hazards regression analyses were performed to assess the independent associations between copper exposure, potential confounding factors, and COPD‐related survival outcomes. Survival analysis was conducted using Kaplan‐Meier curves with Log‐rank tests. The association between copper exposure and COPD risk was evaluated using restricted cubic spline (RCS) models. Pearson's correlation analysis was used to evaluate associations between copper levels and clinical indicators. In addition, subgroup analyses and interaction tests were conducted to evaluate the potential modifying effects of other relevant risk factors on the association between copper levels and acute exacerbations of COPD. Statistical significance was defined as *p* < 0.05.

## 3 Results

### 3.1 NHANES and Clinical Data Confirm That Elevated Copper Levels Are Associated with an Increased Risk of Acute Exacerbation in COPD

**FIGURE 1:**
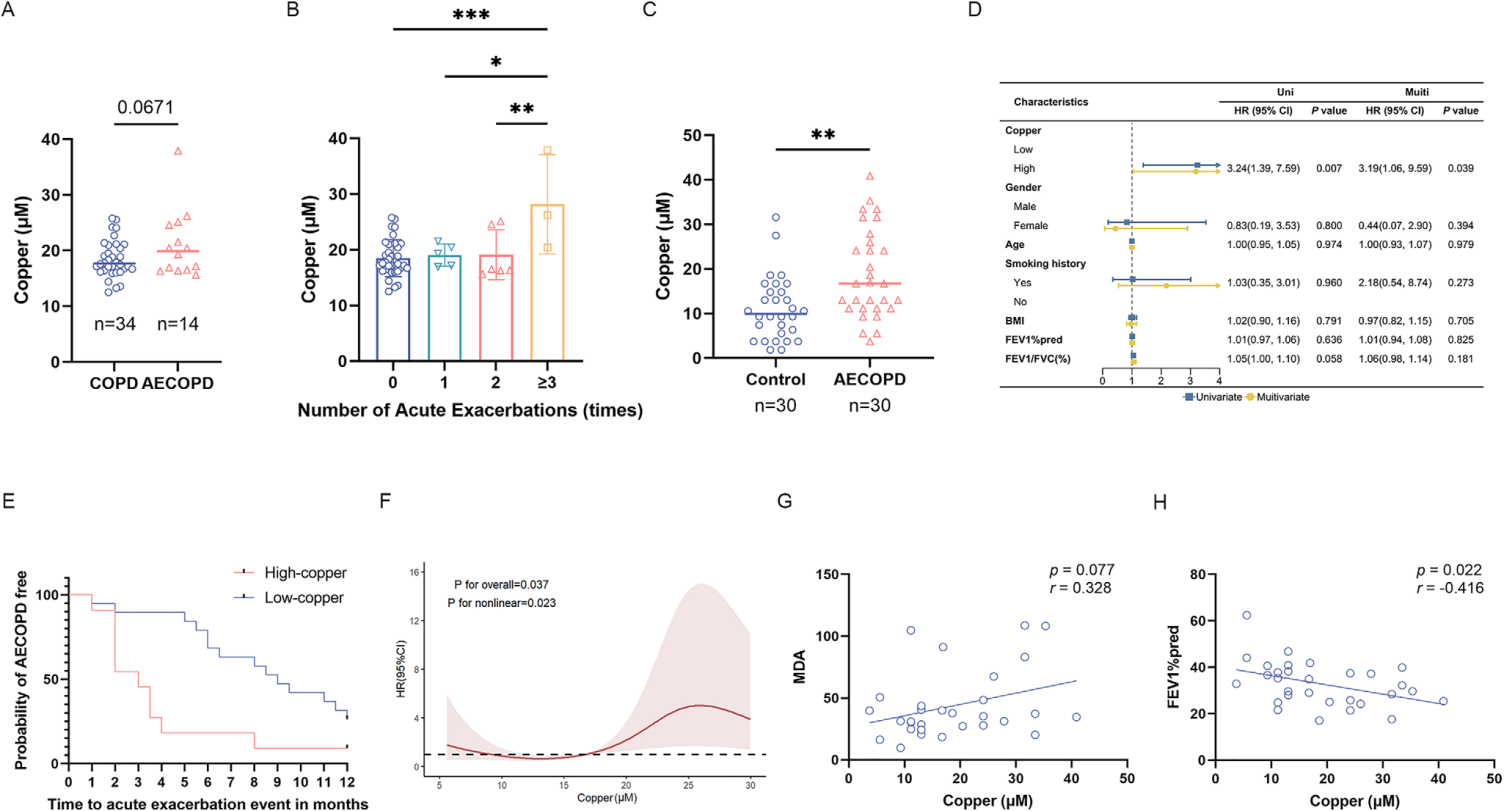
Elevated copper levels correlate with risk of acute exacerbation in COPD. A‐B are based on the NHANES cohort: (A) Serum copper levels of COPD patients in stable phase (n = 34) and acute exacerbation phase (n = 14), Student's t‐test; (B) Serum copper levels in COPD patients with different frequencies of acute exacerbation within one year, ANOVA followed by Tukey's HSD test; C‐H are based on the local cohort: (C) Serum copper levels in AECOPD patients (n = 30) and healthy controls (n = 30), Student's *t*‐test; (D) Forest plot of univariate and multivariate Cox regression analysis; (E) Risk of first post‐discharge acute exacerbation in COPD patients with high or low copper levels; (F) Non‐linear relationship between copper levels and risk of acute exacerbation; G. Correlation between copper levels and lipid peroxidation marker MDA; H. Correlation between copper levels and FEV_1_%pred. (^*^ *p* < 0.05, ^**^ *p* < 0.01, ^***^ *p* < 0.001).

Analysis of NHANES 2011–2012 data revealed significantly higher plasma copper levels in AECOPD patients compared with those with stable COPD (Figure 1A), with the higher levels observed in patients experiencing ≥ 3 exacerbations per year (Figure 1B). In our independent validation cohort (30 AECOPD patients vs. 30 controls; Table S1), serum copper levels were likewise elevated in AECOPD patients (Figure 1C). Cox proportional hazards regression analysis demonstrated that elevated serum copper levels were significantly associated with an increased risk of acute exacerbation in AECOPD patients. This association remained robust after adjustment for age, sex, smoking history, BMI, FEV_1_%pred, and FEV_1_/FVC (Figure 1D). Subgroup analyses showed that the association between serum copper levels and acute exacerbation risk remained consistent across strata of neutrophil percentage (NEU%), C‐reactive protein (CRP), MDA, and GOLD stage, with no significant interactions observed (Figure S1A). Furthermore, during a one‐year follow‐up of 30 COPD patients, higher serum copper levels predicted an earlier first post‐discharge exacerbation (Figure 1E). RCS analysis confirmed a dose‐dependent nonlinear association between serum copper levels and exacerbation risk (likelihood ratio *χ* ^2^ = 8.59, *p* = 0.037) (Figure 1F). The significant spline terms (Copper′: *p* = 0.0075; Copper″: *p* = 0.0068) indicate a nonuniform increase in risk across copper levels. Correlation analysis showed that serum copper levels were positively associated with MDA (*p* = 0.077, Figure 1G) and negatively with FEV_1_%pred (*p* = 0.022, Figure 1H). No significant associations were observed between serum copper levels and other clinical indices (Figure S1B).

### 3.2 In Vivo Models Show That High Copper Exacerbates Emphysema and Epithelial Barrier Damage in COPD Mice

**FIGURE 2:**
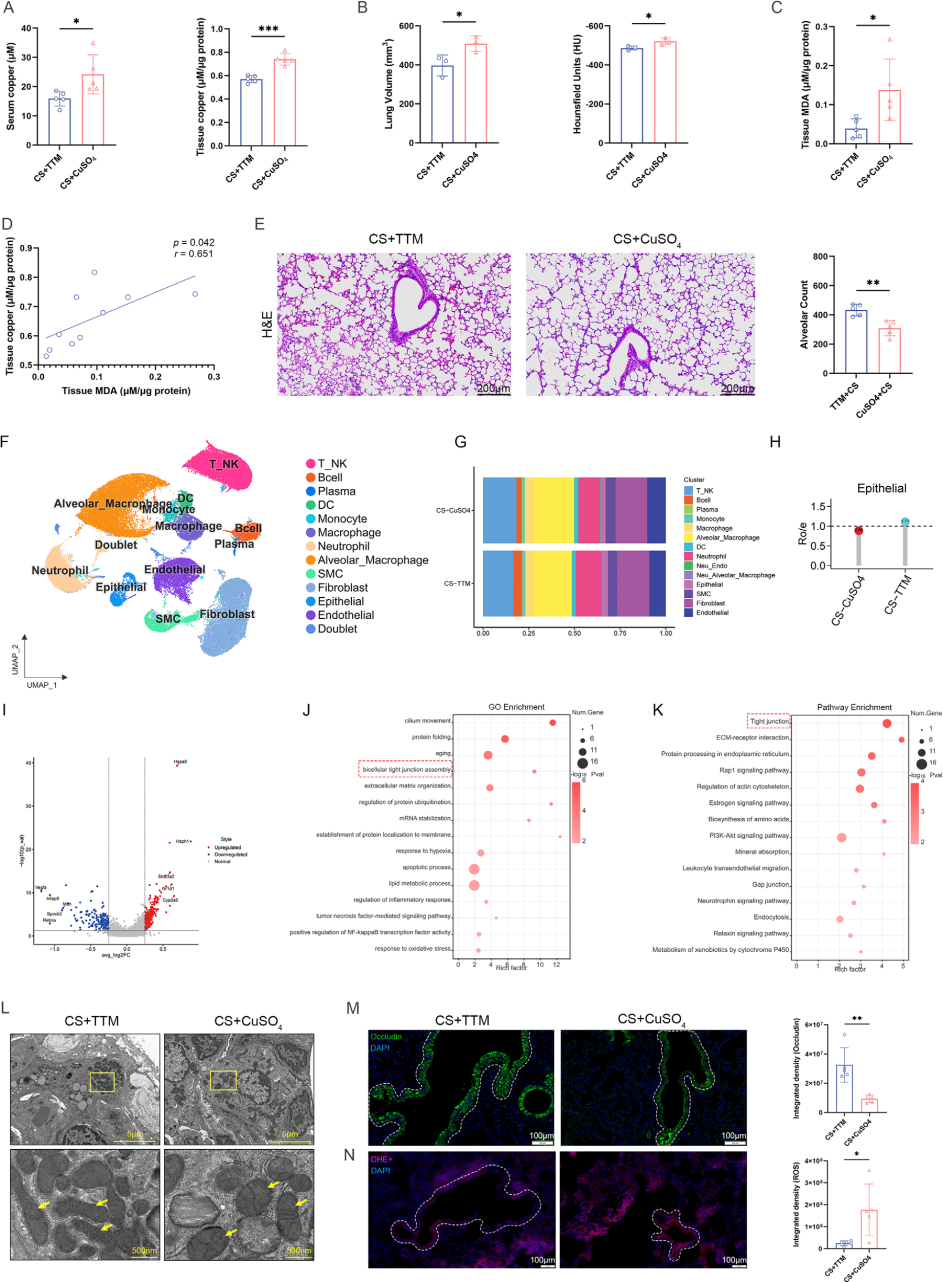
High‐copper environment exacerbates emphysema and epithelial barrier damage in COPD mice. (A) Copper levels in serum and lung tissues of mice; (B) Lung volume and density in mice detected by micro‐CT; (C) MDA levels in mouse lung tissues; (D) Correlation between copper levels and MDA levels in mouse lung tissues; (E) Evaluation of pulmonary emphysema in mice via H&E staining; (F) UMAP plot of cell clustering from single‐cell sequencing of mouse lung tissues (n = 3 per group); (G, H) Proportion of epithelial cells in mouse lung tissues; (I) Volcano plot of DEGs in epithelial cells from mouse lung tissues; (J, K) GO and KEGG enrichment analyses of DEGs; (L) Ultrastructure of mitochondria in mouse pulmonary epithelial cells observed by TEM (Yellow arrows indicate mitochondrial structures); (M) IF staining for tight junction proteins in mouse lung epithelial cells; N. DHE oxidation staining in mouse lung tissues. White dashed circles indicate the airway epithelial regions. (^*^ *p* < 0.05, ^**^ *p* < 0.01, ^***^ *p* < 0.001, n = 5 per group, Student's *t*‐test).

To investigate the role of copper in COPD exacerbation, COPD mice were treated with CuSO_4_ (high copper) or TTM (low copper) to mimic systemic copper variation. High copper exposure significantly increased copper levels in both blood and lung tissues (Figure 2A), increased lung volume, and reduced tissue density as assessed by micro‐CT (Figure 2B), resulting in more severe emphysema (Figure 2E). In contrast, no significant changes were observed in fibrosis or mucus production (Figure S2A, B). Baseline pulmonary function parameters, including FEV_25_/FVC, PEF, and FEF_10_, showed no significant differences between groups (Figure S2C). However, following exposure to H1N1 influenza—a clinically relevant AECOPD—high‐copper COPD mice exhibited marked reductions in these lung function indices compared with low‐copper mice, indicating that copper exposure primes the lung for acute exacerbation in the presence of a secondary insult (Figure S2D). High copper exposure was associated with increased levels of tissue MDA and a higher number of DHE‐positive cells (Figure 2C,N), both of which showed positive correlations with tissue copper levels (Figure 2D). Single‐cell transcriptomic analysis revealed reduced proportions of B cells and epithelial cells in the high‐copper group (Figure 2F–H). Further analysis of airway epithelial subpopulations demonstrated that high copper exposure decreased the proportions of alveolar type 2 (AT2) cells and club cells, while increasing the proportion of ciliated cells (Figure S2E–G). DEGs in airway epithelial cells were significantly enriched in tight‐junction pathways (Figure 2I–K), and IF analysis confirmed reduced expression of tight‐junction proteins (Figure 2M). Corresponding negative controls are shown in Figure S2H. TEM further revealed increased mitochondrial damage under high copper conditions (Figure 2L). Consistent with these findings, zebrafish exposed to 0.1–0.4 µg/mL CuSO_4_ developed dose‐dependent oxidative stress (OS) and progressive gill injury (Figure S3A–C). Collectively, these results demonstrate that systemic copper overload disrupts epithelial barrier integrity and enhances OS, thereby increasing susceptibility to COPD exacerbation.

### 3.3 In Vitro Lung Organoid and BEAS‐2B Models Reveal That Copper Disrupts the Airway Epithelial Barrier via Mitochondria‐OS

**FIGURE 3:**
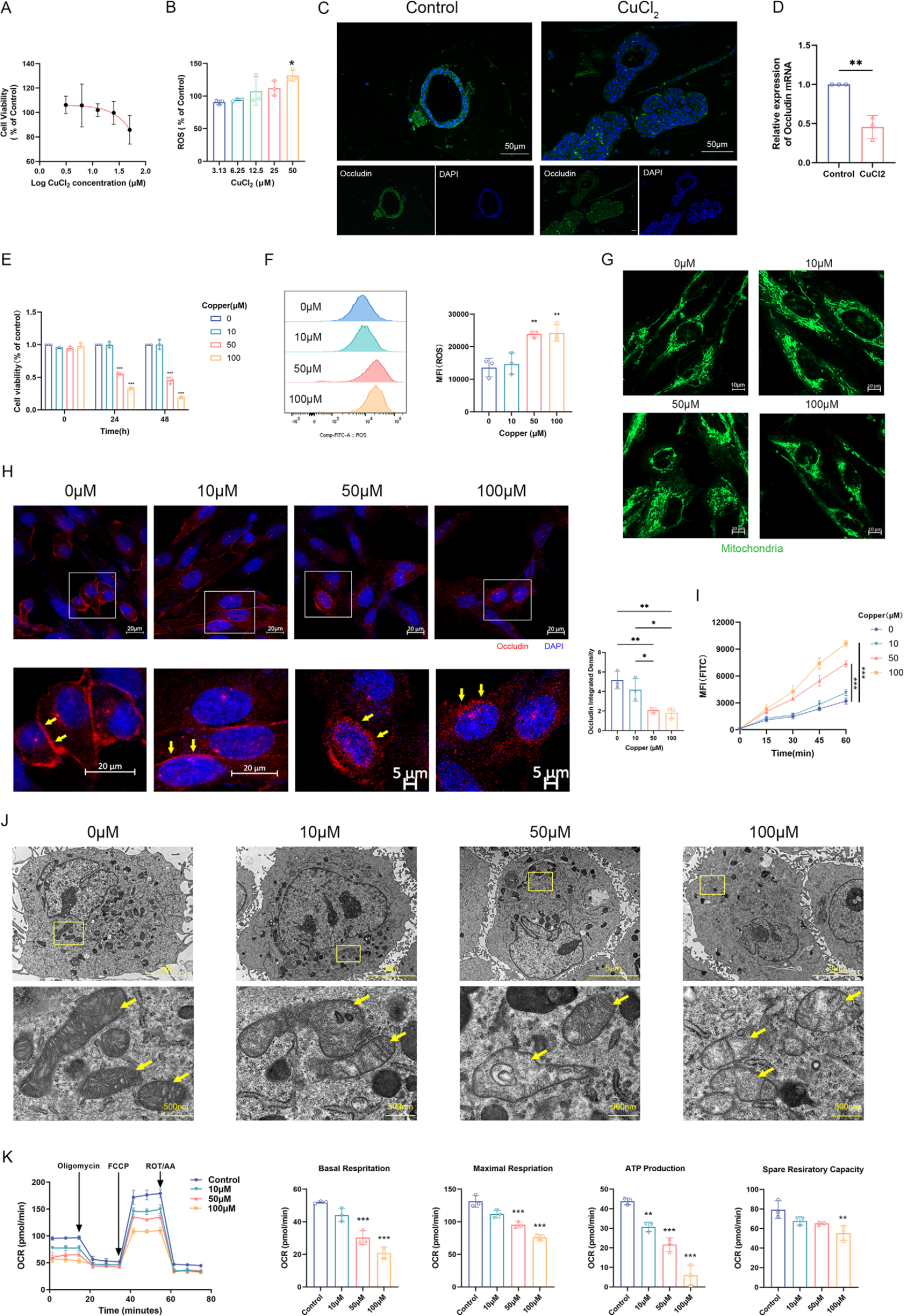
Copper disrupts the airway epithelial barrier via the mitochondria‐oxidative stress axis. (A) Cell viability was detected by ATP content in COPD mouse lung organoids; (B) Effect of CuCl_2_ on ROS levels in COPD mouse lung organoids (compared with control); (C) IF staining for tight junction proteins in COPD mouse lung organoids treated with 50 µm CuCl_2_; D. mRNA levels of tight junction proteins in COPD mouse lung organoids treated with 50 µm CuCl_2_, Student's *t*‐test; (E) Effect of copper on viability of BEAS‐2B cells (compared with 0 µm CuCl_2_); F. Effect of copper on intracellular ROS levels in BEAS‐2B cells (compared with 0 µm CuCl_2_); (G) Effect of copper on mitochondrial morphology and structure; (H) IF staining for tight junction protein expression in BEAS‐2B cells treated with CuCl_2_ (Yellow arrows indicate tight junction protein occludin); (I) Effect of CuCl_2_ on permeability of BEAS‐2B cell monolayers; J. Ultrastructure of mitochondria in BEAS‐2B cells treated with CuCl_2_ observed by TEM (Yellow arrows indicate mitochondrial structures); K. Effect of CuCl_2_ on cellular OCR assessed by Seahorse assay (compared with control). (^*^ *p* < 0.05, ^**^ *p* < 0.01, ^***^ *p* < 0.001, all experiments were performed as three independent replicates, n = 3, ANOVA followed by Tukey's HSD test).

We first established and characterized COPD mouse lung organoids to recapitulate key pathological features of COPD in vitro (Figure S3D, E). To define the cellular composition of organoids, flow cytometry analysis was performed using cell type‐specific markers, showing that epithelial cells (CD326^+^) constituted the largest proportion among CD45^−^ cells (Figure S3G). To evaluate organoid viability following copper exposure, intracellular ATP levels were measured as a well‐established surrogate of metabolic activity and cell viability [29, 30]. CuCl_2_ treatment induced a dose‐dependent reduction in ATP content (Figure 3A), with a significant decrease observed at 50 µm, accompanied by increased oxidative stress (Figure 3B) and disruption of tight junctions (Figure 3C,D). Corresponding negative controls are shown in Figure S3F. Consistent results were obtained in BEAS‐2B cells, in which CuCl_2_ (50–100 µm) reduced cell viability (Figure 3E), increased intracellular ROS levels (Figure 3F), decreased tight‐junction protein expression (Figure 3H, Figure S3H), and increased epithelial permeability (Figure 3I). Mitochondria exhibited fragmentation, swelling, and loss of cristae (Figure 3G,J), along with reduced oxygen consumption (Figure 3K). Collectively, these findings demonstrate that copper‐induced OS and mitochondrial dysfunction impair the integrity of the airway epithelial barrier.

### 3.4 Downregulation of ATP7A Drives Copper Accumulation in COPD Pulmonary Epithelium

**FIGURE 4:**
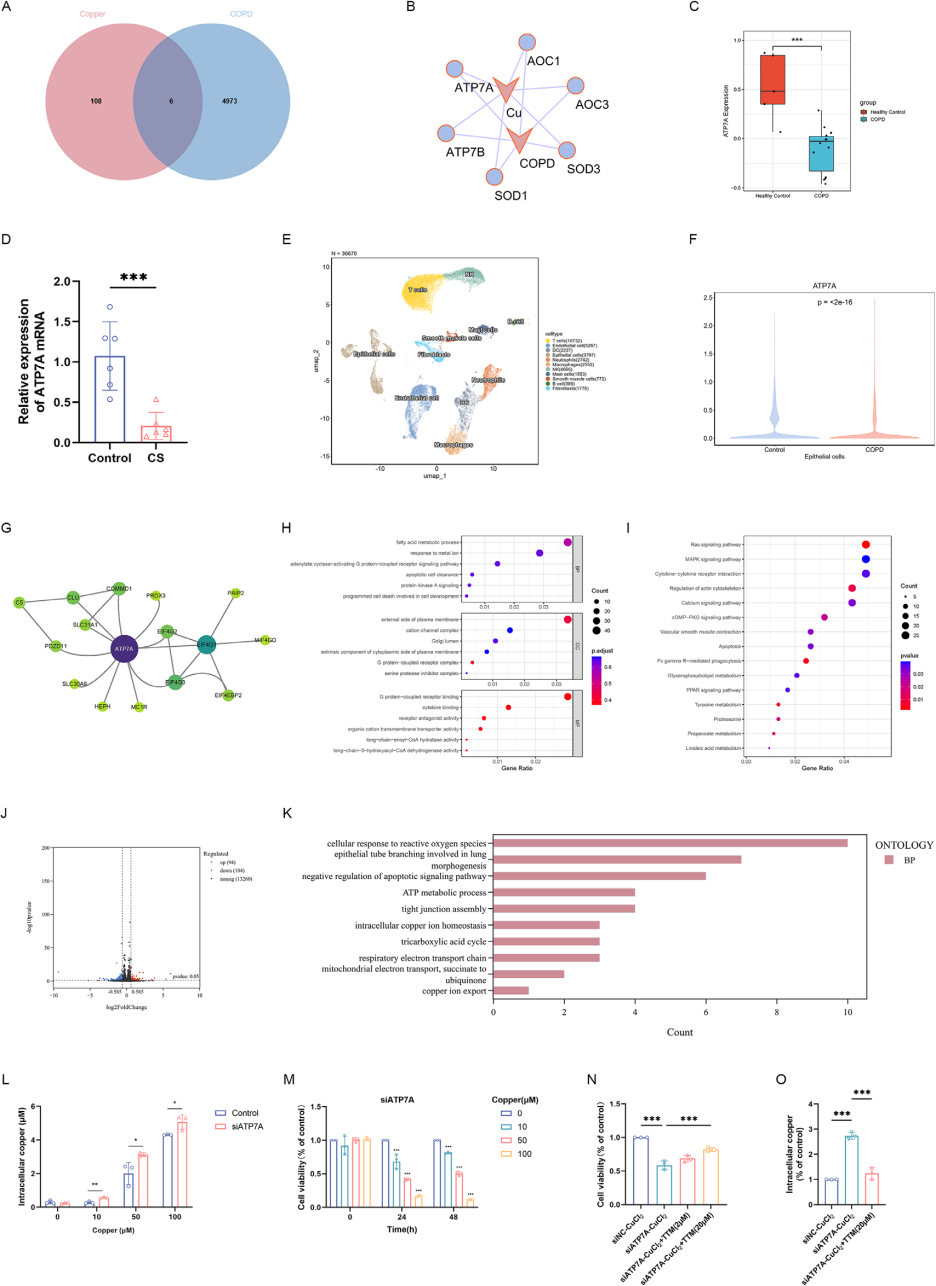
Downregulation of ATP7A drives copper accumulation in COPD lung epithelium. (A, B) Intersection of copper‐related target genes and COPD‐associated target genes; (C) Expression of ATP7A in lung tissues of COPD patients from the GSE106986 dataset; (D) Validation of ATP7A mRNA expression in lung tissues of COPD mice, Student's *t*‐test; (E) UMAP plot of cell clustering from single‐cell sequencing in the GSE173896 dataset; (F) Expression levels of ATP7A in epithelial cells from the GSE173896 dataset; (G) Interaction network between ATP7A and DEGs (GSE106986); (H, I) GO and KEGG enrichment analyses of DEGs in groups with high and low ATP7A expression (GSE106986); (J) Volcano plot of DEGs in BEAS‐2B cells; (K) GO enrichment analysis of DEGs in BEAS‐2B cells; (L) Intracellular copper levels in ATP7A‐knockdown BEAS‐2B cells treated with different concentrations of CuCl_2_; (M) Viability of ATP7A‐knockdown BEAS‐2B cells treated with different concentrations of CuCl_2_ (compared with 0 µm CuCl_2_); (N) Effect of TTM on viability of BEAS‐2B cells with ATP7A knockdown combined with 50 µm CuCl_2_ treatment; (O) Effect of TTM on intracellular copper levels in BEAS‐2B cells with ATP7A knockdown combined with 50 µm CuCl_2_ treatment. (^*^ *p* < 0.05, ^**^ *p* < 0.01, ^***^ *p* < 0.001, all experiments were performed as three independent replicates, n = 3, ANOVA followed by Tukey's HSD test).

To identify factors underlying copper imbalance in COPD, we intersected copper‐related genes from the CTD database with COPD‐associated targets (Figure 4A), yielding six candidate genes (Figure 4B). Among these, ATP7A was the only gene consistently downregulated in both human COPD lung tissue from GSE186986 (Figure 4C, Figure S4A) and COPD mouse lungs (Figure 4D). To further delineate ATP7A expression patterns across lung epithelial cells and their subsets, we analyzed the public single‐cell RNA sequencing dataset GSE173896 (Figure 4E‐F, Figure S4B–F). This single‐cell analysis demonstrated that ATP7A expression was significantly reduced in lung epithelial cells from COPD patients compared with healthy controls, with the most pronounced downregulation observed in AT2 cells, as well as in club cells and ciliated cells. We further performed an in silico ATP7A knock‐in analysis in epithelial cells from the same dataset. Consistent with our experimental findings, virtual restoration of ATP7A expression led to differential regulation of tight‐junction‐related pathways, suggesting a potential protective role of ATP7A in maintaining epithelial barrier integrity (Figure S4G,H). To investigate ATP7A‐related downstream pathways, we stratified samples from GSE106986 into high‐ and low‐ATP7A‐expression groups and identified ATP7A‐associated DEGs, followed by interaction network construction and GO/KEGG enrichment analyses (Figure 4G–I). These DEGs were significantly enriched in pathways related to apoptosis, oxidative stress, tight junctions, and mitochondrial function. In addition, RNA‐seq analysis of ATP7A‐silenced BEAS‐2B cells revealed a distinct DEG profile (Figure 4J, Figure S4I), with GO enrichment again implicating epithelial barrier and stress‐related processes (Figure 4K). GSEA further highlighted autophagy‐related pathways (Figure S4K,L). Functional assays revealed that ATP7A knockdown alone did not alter intracellular copper levels; however, under CuCl_2_ exposure (10–100 µm), ATP7A deficiency markedly increased copper accumulation and reduced cell viability (Figure 4L,M). Collectively, these effects were reversed by treatment with the copper chelator TTM (20 µm) (Figure 4N,O). Collectively, these findings indicate that ATP7A plays a protective role in maintaining epithelial copper homeostasis and that its downregulation increases susceptibility to copper in COPD.

### 3.5 TNF‐α Impairs Copper Homeostasis by Suppressing ATP7A Expression

**FIGURE 5:**
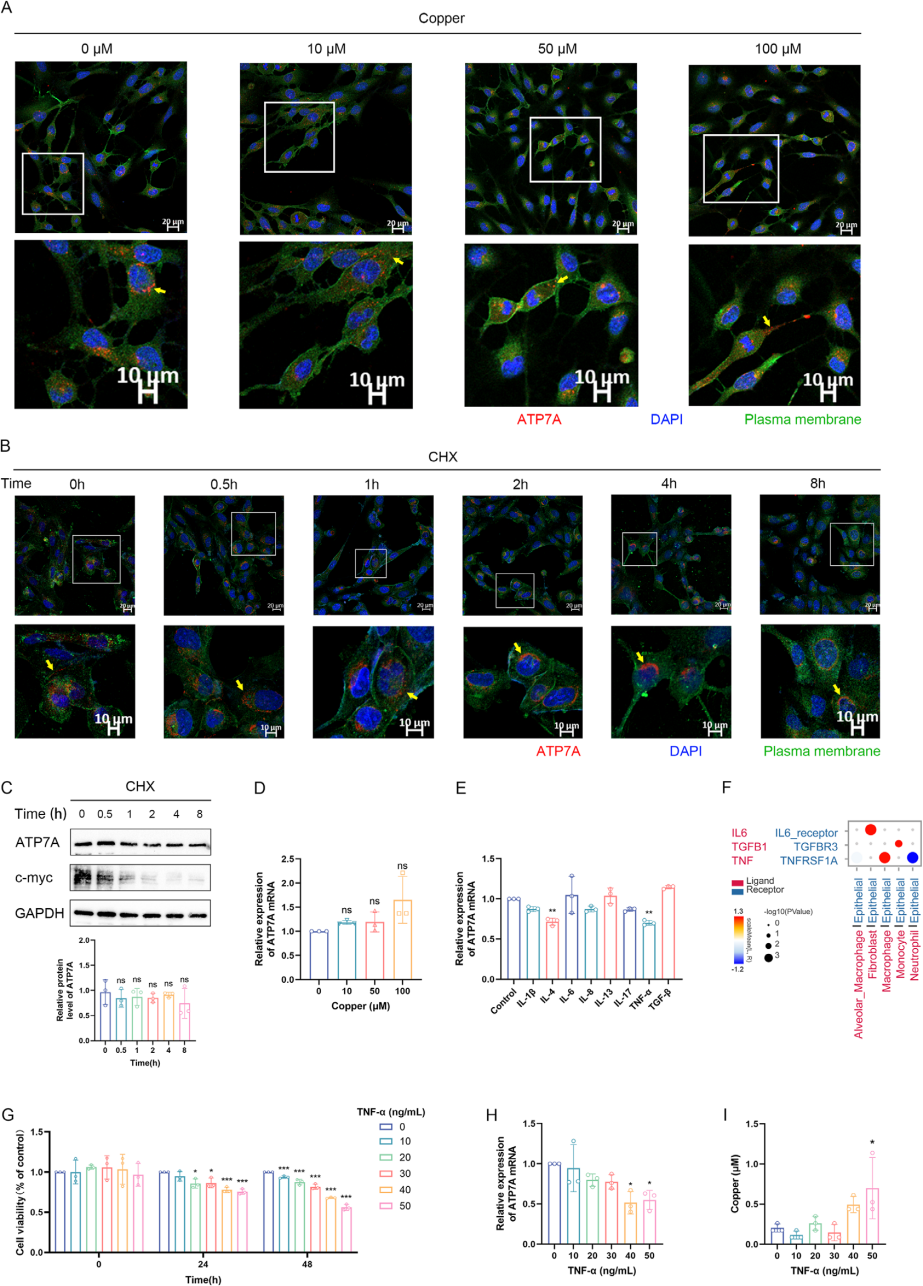
Inflammatory cytokine TNF‐α impairs copper homeostasis by inhibiting ATP7A expression. (A) IF staining for subcellular localization of ATP7A (Yellow arrows show enhanced translocation of ATP7A to the cytoplasmic membrane under elevated CuCl_2_); (B) IF staining for subcellular localization of ATP7A after 100 µg/mL CHX treatment (Yellow arrows indicate time‐dependent translocation of ATP7A from the cytoplasmic membrane toward the trans‐Golgi network); (C) Western blot analysis of time‐dependent expression of ATP7A protein following CHX treatment, with c‐myc as a positive control (compared with 0 µm CuCl_2_); (D) RT‐qPCR analysis of ATP7A mRNA expression in response to various CuCl_2_ concentrations (compared with 0 µm CuCl_2_); (E) RT‐qPCR analysis of ATP7A mRNA expression in response to various cytokines (50 ng/mL each, compared with control); (F) TNF‐α‐TNFRSF1A interaction in epithelial cells; (G) Effect of TNF‐α on cell viability (compared with 0 ng/mL TNF‐α); (H) RT‐qPCR analysis of ATP7A mRNA expression in response to various TNF‐α concentrations (compared with 0 ng/mL TNF‐α); (I) Effect of TNF‐α combined with 10 µm CuCl_2_ on intracellular copper levels (compared with 0 ng/mL TNF‐α). (ns: not significant, ^*^ *p* < 0.05, ^**^ *p* < 0.01, ^***^ *p* < 0.001, all experiments were performed as three independent replicates, n = 3, ANOVA followed by Tukey's HSD test).

To determine the mechanisms underlying ATP7A downregulation in COPD, we first examined its cellular dynamics. Under high‐copper conditions, ATP7A translocated to the plasma membrane to facilitate Cu^2+^ export (Figure 5A). To determine whether the reduced ATP7A expression in COPD is associated with its expulsion from cells or degradation subsequent to copper transport, we treated BEAS‐2B cells with CHX to block new protein synthesis, with c‐myc used as the positive control for the CHX chase assay given its well‐characterized short half‐life and rapid degradation profile. Upon copper withdrawal, ATP7A re‐localized to the trans‐Golgi network (TGN) without significant reduction in total protein levels over an 8 h period (Figure 5B,C); this suggests that ATP7A was neither extruded from cells nor degraded. Furthermore, varying copper concentrations had no effect on ATP7A mRNA expression in BEAS‐2B cells, indicating that elevated copper levels in COPD do not directly modulate ATP7A expression (Figure 5D). We next evaluated COPD‐associated inflammatory mediators and found that among IL‐1β, IL‐4, IL‐6, IL‐8, IL‐13, IL‐17, TNF‐α, and TGF‐β assessed, only IL‐4 and TNF‐α significantly reduced ATP7A mRNA expression (Figure 5E), pointing to specific cytokine‐mediated transcriptional suppression. Notably, single‐cell RNA‐seq analysis revealed that macrophage‐epithelial TNF‐α/TNFRSF1A signaling was specifically enhanced in high‐copper COPD lungs (Figure 5F), which provides a robust rationale for selecting TNF‐α over IL‐4 for further investigation. Consistently, CuCl_2_ treatment induced a dose‐dependent increase in TNF‐α secretion in THP‐1 macrophages (Figure S4M). TNF‐α markedly reduced ATP7A protein levels and cell viability (Figure 5G,H). Moreover, co‐treatment with TNF‐α and 10 µm CuCl_2_ further increased intracellular copper levels (Figure 5I), indicating that chronic TNF‐α exposure—rather than copper itself—is a key driver of ATP7A downregulation and subsequent disruption of copper homeostasis.

### 3.6 ATP7A Overexpression Mitigates TNF‐α and CuCl_2_‐Induced Mitochondrial Dysfunction and Epithelial Barrier Disruption

**FIGURE 6:**
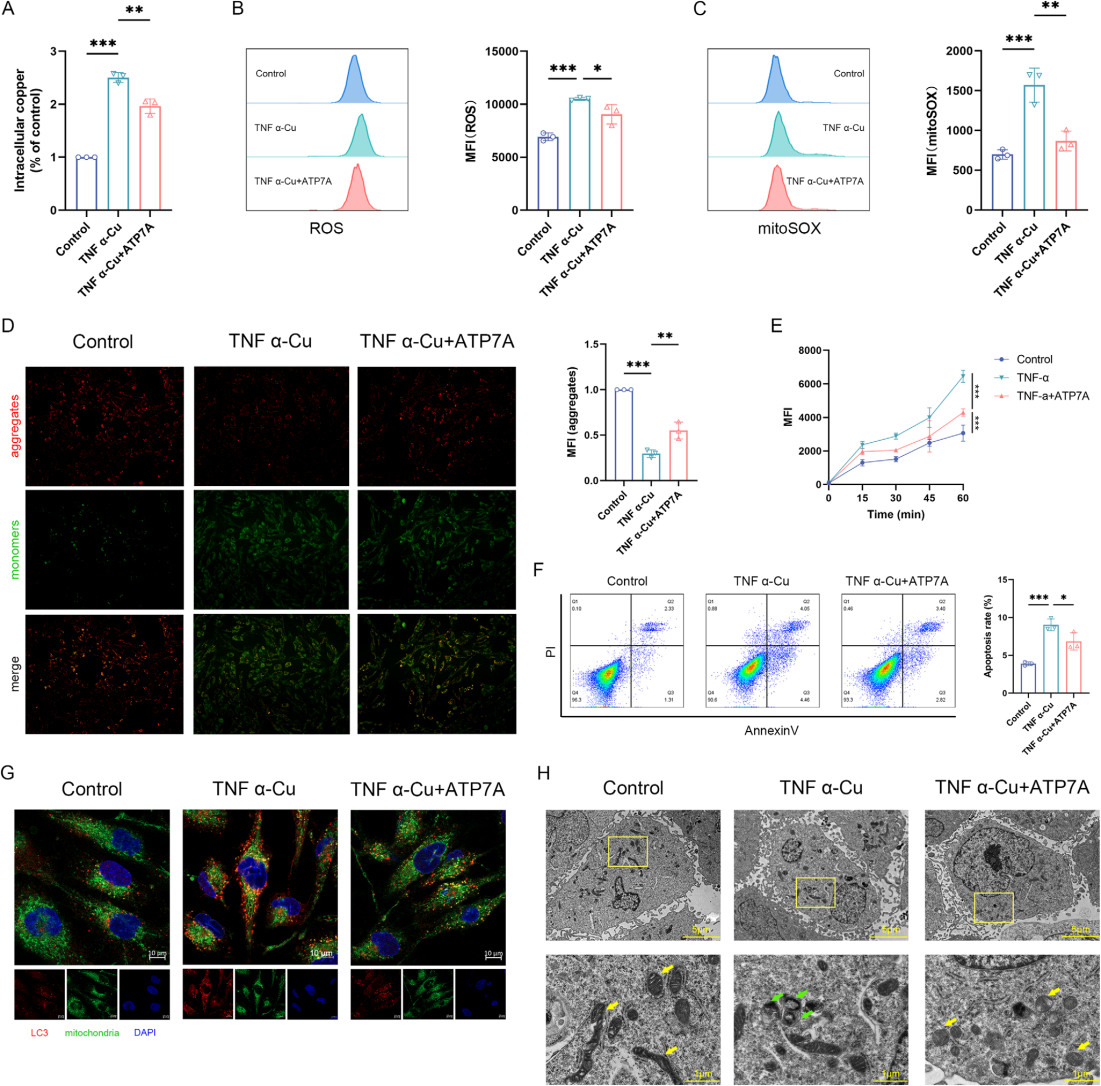
ATP7A overexpression reverses TNF‐α combined with high copper‐induced mitochondrial dysfunction and epithelial barrier disruption. (A) Effect of ATP7A overexpression on intracellular copper elevation induced by TNF‐α‐Cu (TNF‐α: 50 ng/mL + CuCl_2_: 10 µm); (B, C) Effect of ATP7A overexpression on elevated total ROS levels and mtROS levels induced by TNF‐α‐Cu; D. Effect of ATP7A overexpression on reduced mitochondrial membrane potential induced by TNF‐α‐Cu; (E, F) Effect of ATP7A overexpression on increased epithelial permeability and apoptosis in BEAS‐2B cells induced by TNF‐α‐Cu; (G, H) IF staining and TEM observation of the effect of ATP7A overexpression on increased autophagy in BEAS‐2B cells induced by TNF‐α‐Cu. Yellow arrows indicate mitochondria, and green arrows indicate mitophagy in TEM images. (^*^ *p* < 0.05, ^**^ *p* < 0.01, ^***^ *p* < 0.001, all experiments were performed as three independent replicates, n = 3, ANOVA followed by Tukey's HSD test).

BEAS‐2B cells exposed to TNF‐α (50 ng/mL) and CuCl_2_ (10 µm) displayed elevated ROS levels, mitochondrial stress, membrane depolarization, epithelial barrier disruption, apoptosis and mitophagy. Overexpression of ATP7A was validated by RT‐qPCR, which showed a significant increase in ATP7A mRNA expression compared with the control group (Figure S4I). This intervention effectively reduced intracellular copper accumulation (Figure 6A), suppressed OS (Figure 6B,C), restored mitochondrial membrane potential (Figure 6D), repaired barrier integrity (Figure 6E), and decreased apoptosis (Figure 6F) as well as mitophagy (Figure 6G,H).

### 3.7 TNF‐α Suppresses ATP7A Transcription via the NF‐κB/CREB1/CBP Pathway

**FIGURE 7:**
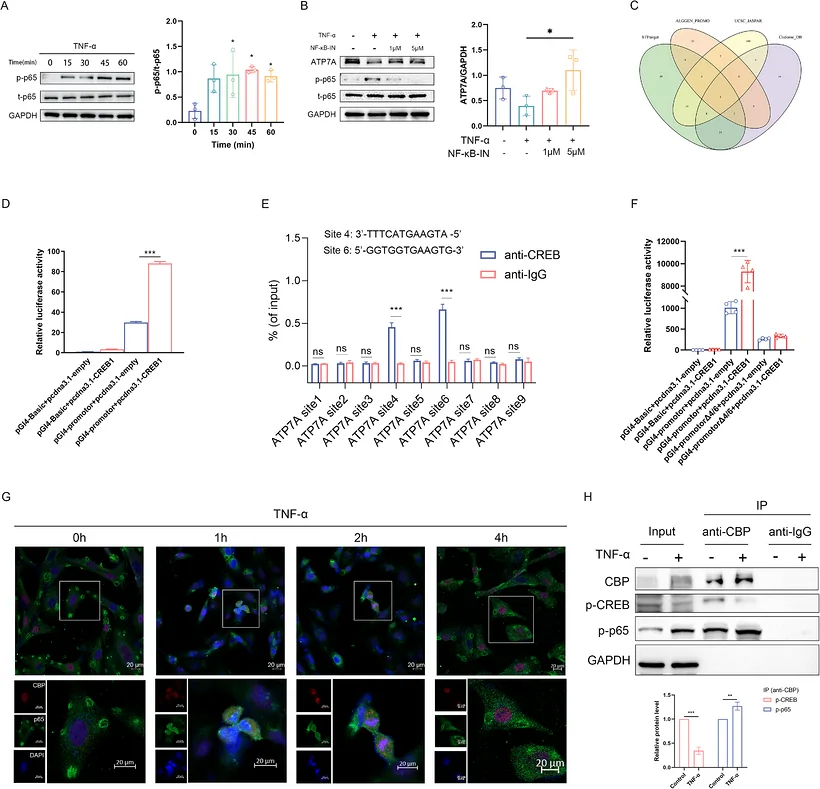
TNF‐α regulates ATP7A transcriptional repression through the NF‐κB/CREB1/CBP. (A) Western blot analysis of time‐dependent expression of phosphorylation p65 protein following 50 ng/mL TNF‐α treatment (compared with 0 min, n = 3); (B) Western blot analysis of phosphorylation p65 and ATP7A protein expression (n = 3); (C) Prediction of transcription factors binding to the ATP7A promoter; (D) Validation of CREB1 binding to the ATP7A promoter via dual‐luciferase reporter assay (n = 3); (E) Validation of CREB1 binding sites in the ATP7A promoter by ChIP‐qPCR (n = 3); (F) Effect of CREB1 binding sites 4/6 deletion on ATP7A promoter activity (n = 4); (G) Nuclear co‐localization of p‐p65 and CBP observed by IF staining; (H) Effect of 50 ng/mL TNF‐α on the binding of p‐p65 and CREB1 to CBP detected by Co‐IP (n = 3). (ns: not significant, ^*^ *p* < 0.05, ^***^ *p* < 0.001, ANOVA followed by Tukey's HSD test).

Previous single‐cell RNA sequencing demonstrated significant enrichment of TNF‐α and NF‐κB signaling in high‐copper COPD mice (Figure 2M). TNF‐α induced phosphorylation of NF‐κB p65 (Figure 7A), and inhibiting p65 nuclear translocation restored ATP7A expression (Figure 7B). However, NF‐κB p65 lacks binding sites in the ATP7A promoter. By integrating four transcription factor databases and performing luciferase assays, CREB1 was identified as an ATP7A activator, and ChIP‐qPCR confirmed its binding at sites 4 and 6 (Figure 7C–E, Figure S5A, Table S2). To functionally validate these findings, dual‐luciferase reporter assays were conducted with targeted deletion of CREB1 binding sites 4 and 6 in the ATP7A promoter, revealing a significant reduction in CREB1 binding affinity (Figure 7F). Molecular docking further illustrated CREB1 at these sites (Figure S5C), and molecular dynamics simulations supported stable CREB1‐DNA interactions (Figure S5D). Mechanistically, p‐p65 and p‐CREB compete for the shared co‐activator CBP (Figure 7G, Figure S5B), and TNF‐α shifted this balance toward p‐p65 and away from p‐CREB (Figure 7H), resulting in transcriptional repression of ATP7A.

### 3.8 Anti‐TNF‐α Therapy Improves Pathological Changes in COPD Mice Through an ATP7A‐Dependent Mechanism

**FIGURE 8:**
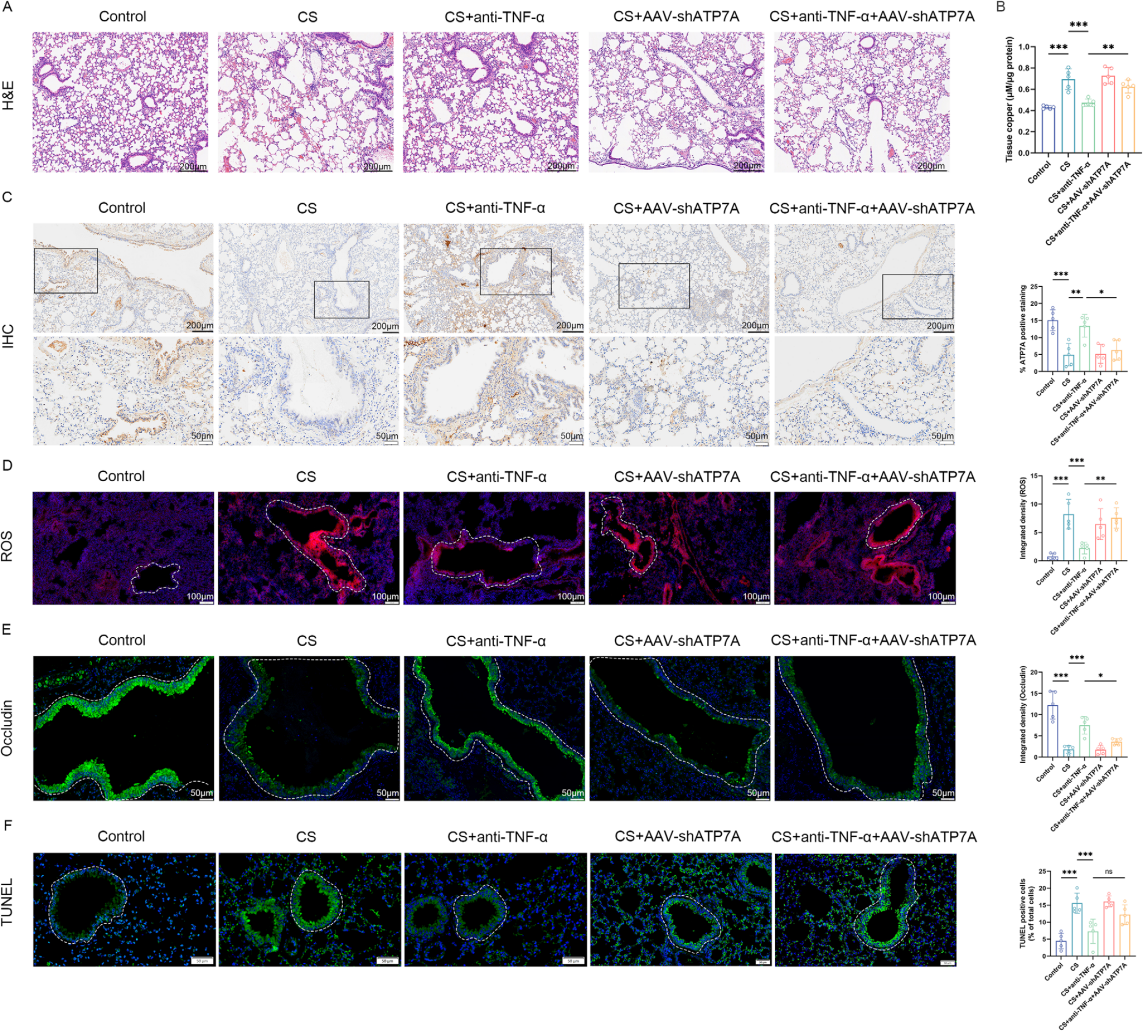
Anti‐TNF α therapy improves pathological changes in COPD mice in an ATP7A‐dependent manner. (A) Evaluation of pulmonary emphysema in mice via H&E staining; (B) Copper levels in mouse lung tissues; (C) IHC staining for ATP7A expressions in mouse lung tissues; (D) DHE oxidation staining in mouse lung tissues; (E) IF staining for tight junction proteins in mouse lung epithelial cells; (F) TUNEL assay for apoptotic cells in mouse lung tissues. White dashed circles indicate the airway epithelial regions. (ns: not significant, ^*^ *p* < 0.05, ^**^ *p* < 0.01, ^***^ *p* < 0.001, n = 5 per group, ANOVA followed by Tukey's HSD test).

**FIGURE 9:**
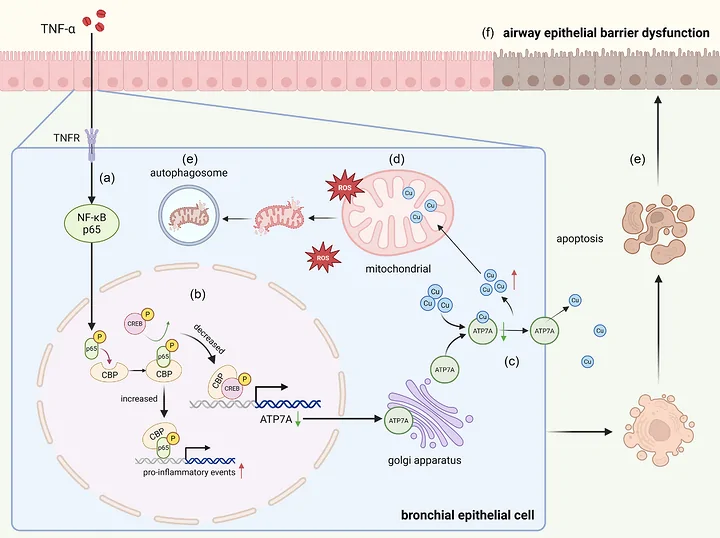
Schematic illustration of the TNF‐α/NF‐κB/CREB1/CBP/ATP7A axis mediating copper accumulation and airway epithelial barrier disruption in AECOPD. (Created with BioRender.com) (a). TNF‐α activates NF‐κB/p65 phosphorylation; (b). Phosphorylated p65 competes with CREB1 for the coactivator CBP, leading to the inhibition of CREB1‐driven ATP7A transcription; (c). Downregulation of ATP7A impairs copper efflux in airway epithelial cells, resulting in intracellular copper accumulation; (d). Excessive intracellular copper induces mitochondrial oxidative stress; (e). Mitochondrial oxidative stress triggers epithelial apoptosis and mitophagy; (f). Epithelial apoptosis and mitophagy cause barrier dysfunction, enhancing susceptibility to pathogenic invasion and inducing AECOPD.

In COPD mice, anti‐TNF‐α treatment (Infliximab, 5 mg/kg i.p., biweekly for 4 weeks) restored ATP7A expression and epithelial tight junction integrity (Figure S6A,B), while reducing BALF TNF‐α and copper levels (Figure S6C,D). Efficient silencing of ATP7A in lung epithelial cells via AAV‐shRNA (Figure 8C) reversed Infliximab‐induced protection against emphysema (Figure 8A) and negated its effects on copper reduction (Figure 8B). Furthermore, the silencing of ATP7A abolished the Infliximab‐mediated decrease in DHE‐positive cells (Figure 8D), reversed its ameliorating effect on barrier dysfunction (Figure 8E), and abrogated its inhibitory action on apoptosis (Figure 8F). Corresponding negative controls are shown in Figure S6E (Figure 9).

## 4 Discussion

This study elucidates how copper imbalance serves as a trigger for AECOPD, revealing a novel mechanistic chain: “high copper—barrier damage—acute exacerbation.” Clinically, analyses of NHANES data and our validation cohort demonstrated that serum copper levels were higher in AECOPD patients than in healthy controls, establishing elevated copper as a measurable risk factor. In animal models, we generated systemic “high copper‐COPD” and “low copper‐COPD” mice. Our results indicated that high copper not only enhanced OS but also disrupted tight junctions and activated mitochondrial apoptosis. Importantly, ATP7A overexpression reversed these detrimental effects, highlighting its potential as a therapeutic target. Cellular experiments further revealed that TNF‐α suppressed ATP7A expression via the NF‐κB/CREB1/CBP competition pathway, leading to copper accumulation and epithelial barrier damage, thereby providing a molecular basis for the observed effects.

Recent studies have confirmed a link between disrupted copper homeostasis and COPD. Elevated copper levels in serum, sputum, and exhaled breath condensate of patients correlate negatively with FEV_1_ [17]. During acute exacerbations, copper levels rise further, suggesting that copper acts as a disease progression accelerator [19]. In addition, large population‐based studies, including analyses from NHANES, have demonstrated a dose‐dependent association between elevated blood copper levels and increased COPD risk, particularly in men [31]. These findings are further supported by cohort evidence showing that higher serum copper levels are associated with an increased risk of developing COPD [21]. Consistent with these published data, our study found that AECOPD patients exhibited higher blood copper levels than stable COPD or healthy controls. Copper levels increased with exacerbation frequency and MDA levels, while decreasing with FEV_1_% pred, underscoring copper imbalance as a measurable clinical factor contributing to OS and lung function decline. However, the relatively small sample size of our study may limit the generalizability of these findings. Future studies should aim to validate these observations in larger cohorts to ensure their reliability and reproducibility.

After establishing systemic “high copper” and “low copper” COPD mouse models, we observed that a high‐copper environment significantly increased OS in lung tissues through lung tissue MDA detection and DHE staining. It should be noted that DHE is not absolutely specific for superoxide anions (O_2_^−^), as its specific product (2‐hydroxyethidium) and non‐specific product (ethidium). Accordingly, MDA provides complementary validation of OS levels to corroborate the DHE results. Copper catalyzes ROS production via Fenton‐like reactions [32], thereby activating inflammatory pathways such as NF‐κB, mitogen‐activated protein kinase (MAPK), and thioredoxin‐interacting protein (TXNIP)‐NOD‐like receptor family pyrin domain‐containing 3 (NLRP3) axis [33, 34]. Excess copper also directly damages mitochondria, disrupts energy metabolism, and aggravates cellular dysfunction [35]. Accumulating evidence has demonstrated that mtROS participates in the regulation of airway epithelial tight junction homeostasis through multiple oxidative stress‐sensitive signaling pathways. For instance, H_2_O_2_‐induced oxidative stress in airway epithelial cells facilitates the degradation of ZO‐1 and claudin‐2 via the TRPM2/PLCγ1/PKCα signaling cascade [36]. Moreover, benzo[a]pyrene has been shown to disrupt airway epithelial tight junctions and impair epithelial barrier integrity through the ROS‐driven NLRP3/caspase‐1 signaling pathway [37]. In addition, ROS can activate the Ca^2+^/MLCK/MLC signaling pathway, thereby inducing the redistribution of occludin and ZO‐1 and ultimately compromising epithelial barrier function [38]. Consistent with these findings, the copper‐induced mitochondrial dysfunction and excessive ROS generation in the present study may impair tight junction integrity and increase epithelial permeability through ROS‐sensitive cytoskeletal and kinase signaling pathways. Notably, because mitochondrial ROS scavengers and pathway‐specific inhibitors were not employed in this study, this mechanistic link should be interpreted as evidence‐supported speculation and requires further experimental validation. In this study, high copper elevated mitochondrial ROS (mtROS) levels and reduced mitochondrial membrane potential. mtROS, a by‐product of oxidative phosphorylation, can modulate cellular homeostasis at moderate levels [39, 40]. However, when mtROS exceeds the antioxidant capacity, it induces OS, damages mitochondrial proteins and metabolic networks, and triggers inflammation and cell death [41, 42], consistent with the apoptosis observed in our high‐copper model. The mitochondrial pathway is central to cell apoptosis, initiated by the Bcl‐2 family through regulation of mitochondrial outer membrane permeability (MOMP) [43]. In the airway epithelium of COPD, the pro‐apoptotic protein Bax is upregulated [44]. Depolarized mitochondria activate Bax, inducing MOMP and cytochrome C release, which in turn triggers the caspase cascade and promotes apoptosis [45]. Therefore, elevated copper not only drives ROS‐mediated cellular damage but also exacerbates COPD tissue injury via mitochondrial apoptosis. A minor consideration is that different soluble divalent copper salts were used in the in vivo and in vitro experiments; although both provide divalent copper ions, their anions could potentially influence copper speciation.

To investigate how systemic copper overload increases the risk of acute COPD exacerbations, we performed single‐cell sequencing on lung tissues from a COPD mouse model exposed to high copper. The results revealed a marked reduction in the proportion of epithelial cells. The airway epithelium serves as the first line of defense against pathogens and environmental pollutants [46, 47], and its damage increases susceptibility to infection and inflammation, thereby contributing significantly to acute COPD exacerbations [48]. Functional enrichment analysis further showed that DEGs between high‐copper and low‐copper COPD mice were strongly associated with the “tight junction” pathway. Tight junctions, composed of transmembrane and cytoplasmic scaffolding proteins, are critical for maintaining epithelial barrier integrity and for protecting the airway by regulating permeability, immune responses, and tissue repair [49, 50]. Elevated copper disrupts these junctions, facilitating pathogen penetration and triggering secondary infections, which in turn promote acute exacerbations. In summary, systemic copper overload compromises the epithelial barrier—particularly by impairing tight junctions—thereby creating a conduit for pathogen entry and increasing the risk of acute COPD exacerbations.

Copper homeostasis imbalance plays a central role in driving inflammation and OS in COPD [18, 33]. Notably, while TTM exhibits robust efficacy in targeting copper, it also presents potential off‐target effects that necessitate thorough evaluation. Specifically, high‐dose and long‐term administration of TTM have been shown to elicit adverse reactions associated with copper deficiency, such as myelosuppression, neurotoxicity, and impaired angiogenesis. These findings underscore the limitations of direct copper‐chelating agents in clinical applications and highlight the need for alternative strategies. Consequently, the modulation of copper‐related genes emerges as a promising therapeutic avenue for COPD, potentially circumventing the challenges posed by conventional copper‐chelation therapies. In line with this notion, accumulating evidence indicates that copper‐related genes are dysregulated in COPD lung tissue: the copper importer CTR1 is upregulated in smoking‐related models, while Dihydrolipoamide dehydrogenase (DLD) and cyclin‐dependent kinase inhibitor 2A (CDKN2A) correlate with disease severity, potentially exacerbating lung injury through mitochondrial dysfunction and cell cycle disruption [51]. Dysfunction of the copper‐dependent enzyme lysyl oxidase (LOX) has been directly linked to emphysematous changes [52]. Cuproptosis, a form of copper‐induced mitochondrial cell death, provides a novel mechanism underlying epithelial loss in COPD [53, 54]. Notably, the cuproptosis‐related gene glutaminase is downregulated in COPD alveolar macrophages, promoting copper accumulation and macrophage death [55]. Collectively, genes involved in copper homeostasis may serve as potential biomarkers and support targeting copper metabolism as a therapeutic strategy. Our study further identifies ATP7A as a “gatekeeper” of epithelial copper homeostasis in COPD, regulating copper efflux and modulating OS and inflammation.

Although ATP7B is also an important copper‐exporting ATPase and may contribute to epithelial copper homeostasis, it is mainly involved in hepatic biliary copper excretion and systemic copper regulation. In contrast, ATP7A is broadly expressed in extrahepatic tissues and was therefore prioritized in this study. ATP7A is a transmembrane copper transporter primarily located in the TGN, where it delivers copper to copper‐dependent enzymes in the secretory pathway. Upon elevation of extracellular copper, ATP7A traffics to the plasma membrane to facilitate copper efflux [56, 57, 58, 59, 60]. In this study, we observed that following copper efflux, ATP7A returned to the TGN without undergoing ubiquitination‐mediated degradation, demonstrating its capacity for recycling. This finding raises questions regarding the factors in the COPD microenvironment that lead to ATP7A downregulation. Stimulation of BEAS‐2B cells with various inflammatory mediators revealed that only TNF‐α markedly suppressed ATP7A expression. CHX chase experiments further confirmed that copper itself does not destabilize ATP7A nor alter its mRNA levels, indicating that ATP7A downregulation in COPD results from TNF‐α‐mediated transcriptional repression rather than copper‐induced protein degradation. Moreover, TNF‐α/TNFRSF1A signaling was enhanced in epithelial cells of high‐copper COPD mice, and THP‐1 macrophage experiments confirmed that copper exposure stimulates TNF‐α secretion, establishing a self‐reinforcing feedback loop: copper accumulation induces macrophages to release TNF‐α, which inhibits ATP7A in epithelial cells, impairs copper efflux, exacerbates copper retention and inflammation, and amplifies mitochondrial oxidative stress and epithelial barrier dysfunction, thereby promoting AECOPD progression. These findings are consistent with Liu et al. [61], who reported that TNF‐α overexpression in mice reduced levels of ATP7A, ATP7B, and copper‐dependent enzymes such as AOC3 and LOX, further supporting the conclusion that TNF‐α disrupts pulmonary copper homeostasis by impairing the copper transport system.

We recapitulated the “high TNF‐α + high copper” COPD microenvironment by co‐stimulating BEAS‐2B cells with both factors. ATP7A overexpression reduced intracellular copper accumulation and mitigated multiple TNF‐α‐Cu‐induced pathologies, including OS, mitochondrial dysfunction, epithelial barrier disruption, apoptosis, and mitophagy. Mechanistic investigations using single‐cell sequencing revealed enrichment of the NF‐κB pathway in epithelial cells under high copper, a well‐known TNF‐α target. Although inhibitors of p65 nuclear translocation restored ATP7A expression, no p65 binding sites were detected in the ATP7A promoter, excluding direct transcriptional repression. By integrating four transcription factor databases with experimental assays, we identified CREB1 as a critical regulator. TNF‐α‐activated p65 suppressed ATP7A by competing with CREB1 for the shared coactivator CBP, thereby inhibiting CREB1‐driven transcription. These findings are consistent with previous studies demonstrating that NF‐κB/CREB competition for CBP modulates inflammation and other cellular processes across multiple models [62, 63, 64, 65]. This conserved “inflammatory switch” may represent a unifying therapeutic target for diverse inflammatory diseases.

Our animal experiments further demonstrated that anti‐TNF‐α therapy reduced copper accumulation and OS in the lungs of COPD mice, restored tight‐junction protein expression, and inhibited epithelial cell apoptosis. However, these protective effects were abolished by ATP7A knockdown, indicating that ATP7A is a critical mediator of the therapeutic effects of TNF‐α blockade. Nevertheless, our in vivo evidence primarily relied on ATP7A knockdown to demonstrate its necessity, whereas ATP7A overexpression rescue was not performed in animal models. Future studies using animal rescue models are needed to further validate the causal role of ATP7A in epithelial barrier protection and AECOPD progression. Previous studies using COPD models induced by cigarette smoke or lipopolysaccharide have shown that anti‐TNF‐α antibodies reduce alveolar macrophage and neutrophil infiltration, decrease inflammatory cytokines such as IL‐6 and IL‐8, and attenuate emphysema and airway remodeling. Nevertheless, clinical trial outcomes have been inconsistent. Randomized trials reported no significant improvements in lung function, quality of life, or exacerbation frequency with Infliximab or Etanercept, and Infliximab was associated with an increased risk of infection [66, 67, 68]. These clinical shortcomings necessitate a mechanistic and strategic reappraisal rather than outright dismissal of TNF‐α as a therapeutic target.

One plausible explanation for these inconsistent outcomes is the pronounced heterogeneity of COPD, which may limit the effectiveness of nonselective therapeutic approaches. Accordingly, an increasing number of studies have sought to capture COPD heterogeneity through quantitative, data‐driven approaches for more refined patient stratification. For example, Yin et al. developed a fractional dynamics deep learning model that extracts fractional‐order signatures from physiological signals, including thoracic breathing effort, respiratory rate, and oxygen saturation, to distinguish different COPD stages with high accuracy [69]. Other recent approaches have integrated routinely collected clinical variables using machine‐learning algorithms to predict near‐term AECOPD risk [70], whereas quantitative CT features combined with deep learning have been used to stratify patients according to their future exacerbation risk [71]. Collectively, these studies demonstrate that COPD heterogeneity can be captured across physiological, clinical, and imaging dimensions and highlight the potential value of more refined patient stratification. However, these predictive approaches primarily stratify patients according to disease severity or exacerbation risk, whereas they do not directly resolve the biological mechanisms that may underlie differential therapeutic responsiveness. In this context, our findings suggest a candidate mechanism‐based endotype characterized by elevated TNF‐α, ATP7A suppression, copper accumulation, mitochondrial oxidative stress, and epithelial barrier disruption. This concept is supported by clinical observations suggesting the existence of a TNF‐responsive subgroup. Post–hoc analyses of infliximab trials revealed a trend toward improved 6 min walk distance in younger or cachectic patients. Moreover, an observational study reported a 51% reduction in COPD‐related hospitalization among patients receiving TNF‐α inhibitors—particularly etanercept—for concomitant rheumatoid arthritis [72]. In addition, the biological activity of TNF‐α may be phase‐dependent. Evidence indicates that during stable COPD, circulating TNF‐α and its soluble receptors are not consistently elevated and show weak correlations with disease markers. In contrast, TNF‐α levels rise markedly during acute exacerbations and are implicated in initiating inflammatory cascades [73]. Accordingly, the therapeutic window for anti‐TNF‐α strategies may be more relevant for preventing or attenuating exacerbations rather than modifying stable airflow limitation, which aligns directly with our focus on AECOPD pathogenesis. Based on these findings, we propose that individuals exhibiting both elevated copper levels and high TNF‐α expression may represent a candidate subgroup more likely to benefit from anti‐TNF‐α therapy. In conclusion, the failure of TNF‐α inhibitors in unselected COPD populations should not lead to abandonment of this pathway but should instead motivate a shift toward precision medicine. Future studies should validate this biomarker‐guided stratification strategy in clinical cohorts and evaluate the safety and preliminary efficacy of anti‐TNF‐α therapy in COPD patients characterized by concurrent copper overload and TNF‐α activation.

This study demonstrates that copper dyshomeostasis, driven by the TNF‐α/ATP7A axis, plays a critical role in acute exacerbations of COPD. Elevated copper levels in COPD patients contribute to epithelial barrier disruption by inducing mitochondrial dysfunction and OS in airway epithelial cells. TNF‐α suppresses ATP7A expression through NF‐κB/CREB1/CBP competition, thereby impairing copper efflux and exacerbating epithelial injury(Figure 9). Collectively, these findings provide both a theoretical and experimental basis for the development of precision therapeutic strategies, including copper chelators, ATP7A activation, or targeted modulation of NF‐κB/CREB1 crosstalk.

## Author Contributions

**Xinru Xiao**: Writing – original draft, Investigation, Validation, Conceptualization; **Yongzhe Hao**: Investigation, Validation; **Ziqi Ding**: Validation, Data curation; **Zhipeng Wang**: Validation, Data curation; **Yujia Shi**: Visualization; **Yanhua Huang**: Visualization; **Chunjian Qi**: Writing – review & editing, Supervision, Conceptualization; **Qian Zhang**: Writing – review & editing, Project administration, Funding acquisition, Conceptualization.

## Funding

This study was supported by grants from the Noncommunicable Chronic Diseases‐National Science and Technology Major Project (2024ZD0524000 to Q.D.), the Changzhou High‐Level Medical Talents Training Project (2022CZLJ013 to Q.Z.), the Development Plan of Traditional Chinese Medicine Science and Technology in Jiangsu Province (MS2024075 to Q.Z.), the Changzhou Sci & Tech Program (CJ20241117 to Q.Z.), and the Research Project of Changzhou Medical Center of Nanjing Medical University (CMCC202303 and CMCB202405 to Q.Z.).

## Ethics Approval

All participants provided informed consent for sample use in research, and the study was approved by the Ethics Committee of the Third Affiliated Hospital of Nanjing Medical University ([2024]KY244‐01). All experimental procedures were approved by the Nanjing Medical University Laboratory Animal Ethics Committee (Approval ID: IACUC24‐0110).

## AI Use Statement

No generative artificial intelligence or AI‐assisted technologies were used in the writing process of this manuscript.

## Conflicts of Interest

The authors declare no conflict of interest.

## Supporting information




**Supporting File**: advs77977‐sup‐0001‐SuppMat.docx.

## Data Availability

The data that support the findings of this study are available in the supplementary material of this article.
