# Supplementary material for: Copper‐Driven Epithelial Barrier Disruption: A Novel Mechanism of COPD Acute Exacerbations Mediated by the TNF‐α/ATP7A Axis
Source: Adv Sci (Weinh). 2026 Sep 27:e19533. Online ahead of print. doi: 10.1002/advs.202519533 (PMC13616358; doi:10.1002/advs.202519533)
Supplement: Supplementary file 1 [file ADVS-9999-e19533-s001.docx]

**Supplementary Figure 1. Subgroup analysis and correlation analysis of serum copper levels with clinical indices in COPD patients**

A. Subgroup analysis for acute exacerbation risk; B. Correlations with clinical parameters.

BMI: Body mass index, WBC: white blood cell count, EOS: eosinophil count, NEU%: neutrophil percentage, LDH: lactate dehydrogenase, CRP: C-reactive protein, ESR: erythrocyte sedimentation rate, PCT: procalcitonin, pH: hydrogen ion concentration, PaO_2_: partial pressure of oxygen in arterial blood, PaCO_2_: partial pressure of carbon dioxide in arterial blood, HCO_3_⁻: bicarbonate, BE: base excess, SaO_2_: arterial oxygen saturation.

**Supplementary Figure 2. Effects of copper in COPD models on lung histology and airway epithelial subpopulation composition**

A. MASSON staining analysis of lung collagen deposition; B. PAS staining for lung mucus secretion; C. Lung function parameters (FEV_25_/FVC, PEF, FEF_10_) in mice; D. Lung function parameters (FEV_25_/FVC, PEF, FEF_10_) in mice following H1N1 infection; E. UMAP plot showing four airway epithelial subpopulations in COPD models; F. Heatmap of cell-type marker gene expression; G. Quantitative analysis of epithelial subpopulation proportion; H. Negative control sections stained without primary antibody. (ns: not significant, ***P* < 0.01, n = 5 per group, Student’s t-test)

**Supplementary Figure 3. Zebrafish toxicity assays and lung organoid characterization**

A. Mortality rate of zebrafish exposed to CuSO_4_ (n = 8 per group); B. Oxidative stress levels in zebrafish exposed to CuSO_4_; C. Morphological changes in gill epithelial cells of CuSO_4_ exposed zebrafish; D-E. Construction and validation of lung organoids from COPD mice; F. Universal negative control for all organoid experimental groups, incubated without primary antibody and only stained with DAPI and Cy3-labeled Goat Anti-Rabbit IgG; G. Flow cytometry analysis reveals the cellular composition ratio of lung organoids (n = 3); H. Universal negative control for all cell experimental groups, incubated without primary antibody and only stained with DAPI and Cy3-labeled Goat Anti-Rabbit IgG. (ns: not significant, ****P* < 0.001, ANOVA followed by Tukey's HSD test)

**Supplementary Figure 4. ATP7A expression, regulation, and functional effects**

A. Expression of SOD1, SOD3, ACO3, and ATP7B (ACO1 was excluded due to extremely low expression) in lung tissues of COPD patients from the GSE106986 dataset; B-F. Cell subpopulation and ATP7A expression (GSE173896 dataset): (B) Marker gene expression of major lung cell populations; (C) ATP7A expression across cell populations; (D) UMAP plot of airway epithelial subpopulations; (E) Marker gene heatmap of epithelial subpopulations; (F) ATP7A expression in epithelial subpopulations. G-H. GO and KEGG enrichment analyses of DEGs after in silico ATP7A knock-in in epithelial cells from the GSE173896 dataset; I. Validation of ATP7A overexpression, Student’s t-test; J. Validation of ATP7A knockdown efficiency (compared with siNC); K. GSEA of transcriptome sequencing data comparing ATP7A-knockdown and control BEAS-2B cells; L. GSEA enrichment analysis of dataset GSE106986 in groups with high vs. low ATP7A expression; M. TNF-α secretion in THP-1 cells treated with CuCl_2_.(ns: not significant, **P* < 0.05, ***P* < 0.01, ****P* < 0.001, all experiments were performed as three independent replicates, n = 3, ANOVA followed by Tukey's HSD test)

**Supplementary Figure 5. CREB1-ATP7A promoter binding design and molecular interactions**

A. Schematic of primer design sites for ChIP-qPCR targeting CREB1 binding to the ATP7A promoter; B. Universal negative control for all cell experimental groups, incubated without primary antibody and only stained with DAPI, Cy3-labeled Goat Anti-Rabbit IgG and FITC-labeled Goat Anti-Mouse IgG; C-D. Molecular docking and molecular dynamics simulations investigating the binding stability between CREB1 and the ATP7A promoter.

**Supplementary Figure 6. Anti-TNF α therapy improves pathological changes in COPD mice**

A. IHC staining for ATP7A expressions in mouse lung tissues; B. IF staining for tight junction proteins in mouse lung epithelial cells; C. TNF-α concentrations in BALF of mice; D. Copper levels in BALF of mice; E. Negative control sections stained without primary antibody. (**P* < 0.05, ***P* < 0.01, ****P* < 0.001, n = 5 per group, ANOVA followed by Tukey's HSD test)

**Supplementary Table 1. Characteristics of patients**

| **Variable** | **Healthy**  **(*n* = 30)** | **AECOPD**  **(*n* = 30)** | ***P* value** |
| --- | --- | --- | --- |
| **Gender (female/male)** | 4/26 | 3/27 | 0.688 |
| **Age (years)** | 67.80±0.85 | 70.23±1.18 | 0.100 |
| **BMI (kg/m^2^)** | 22.50±0.34 | 21.53±0.61 | 0.168 |
| **Smoking history (no/yes)** | 6/24 | 5/25 | 0.739 |
| **FEV_1_/FVC** | 101.35 (93.83, 111.75) | 34.82 (30.93, 46.59) | < 0.001 |
| **FEV_1_%pred** | 84.40 (79.62, 88.25) | 32.55 (25.33, 40.08) | < 0.001 |
| **WBC** | 5.89 (5.58, 7.33) | 8.68 (6.81, 12.82) | < 0.001 |
| **EOS** | 0.09 (006, 0.15) | 0.12 (0.03, 0.21) | 0.651 |
| **NEU%** | 62.35 (53.38, 67.18) | 70.95 (61.10, 79.68) | 0.001 |

Data are presented as mean ± SEM for normally or approximately normally distributed data, and as median (interquartile range) [M (P25, P75)] for non-normally distributed data.

**Supplementary Table 2. ChIP-qPCR Primer Sequences**

| Gene Name | Primer Sequence (5’-3’) |
| --- | --- |
| ATP7A primer 1F  ATP7A primer 1R | CTACTTGGGAGGCTGAGGTG  TTTGGTTTTGAGAGTCTTGCTG |
| ATP7A primer 2F  ATP7A primer 2R | TTTTTGGTTTTGCCTTTTAGTGA  TGTCAATGTGTGCAGCCTTA |
| ATP7A primer 3F  ATP7A primer 3R | TGTTGCCTGTCAAGGCTTAAT  TGAAGCCTAATAATGTTGCAGAA |
| ATP7A primer 4F  ATP7A primer 4R | TGTGGGAACTGGGTTCAAAT  AAAAAGAGGGAGAGGCTCCA |
| ATP7A primer 5F  ATP7A primer 5R | CCTAGAATTTTGCTGCTTTTCAA  GATTCCGGTGATGCAAAAGT |
| ATP7A primer 6F  ATP7A primer 6R | CAGAGACTGTAACACTTTTGCATCA  TTTTATATGTGTGTTGAGGGGGTA |
| ATP7A primer 7F  ATP7A primer 7R | CCTACCCCCTCAACACACAT  TGTGTTAACTTGCTTCCCGTA |
| ATP7A primer 8F  ATP7A primer 8R | CCCTAACAAAGACCCACTCG  AAAGAGAGCGACTGGTCACG |
| ATP7A primer 9F  ATP7A primer 9R | TCTAGACCACGCGTTCCTTT  CTGCTTCTCTTTTCCCACCC |
| GAPDH | AAAAGCGGGGAGAAAGTAGG  AAGAAGATGCGGCTGACTGT |
